# Plasma calprotectin level: usage in distinction of uncomplicated from complicated acute appendicitis

**DOI:** 10.1186/s13017-016-0062-9

**Published:** 2016-01-27

**Authors:** Murat Cikot, Kivanc Derya Peker, Mehmet Abdussamet Bozkurt, Ali Kocatas, Osman Kones, Sinan Binboga, Asuman Gedikbasi, Halil Alis

**Affiliations:** Department of General Surgery, Istanbul Bakirkoy Dr. Sadi Konuk Training and Research Hospital, Zuhuratbaba Mh, Tevfik Saglam Cad. No: 11, 34147 Bakirkoy/Istanbul, Turkey; Department of Biochemistry, Istanbul Bakirkoy Dr. Sadi Konuk Training and Research Hospital, Bakirkoy/Istanbul, Turkey

**Keywords:** Acute appendicitis, Plasma Calprotectin, Uncomplicated acute appendicitis, Complicated acute appendicitis

## Abstract

**Background:**

The aim of this study was to identify the diagnostic role of plasma calprotectin value for a distinction of presence acute appendicitis and the indifference of uncomplicated from complicated acute appendicitis.

**Methods:**

Plasma calprotectin, white blood cell and C-reactive protein values of 89 patients, who have undergone laparoscopic appendectomy between January 2013 and May 2013 were evaluated.

**Results:**

Calprotectin was 91 ng/mL (range 45–538) for acute appendicitis and 47 ng/ml (range 28–205) for the control group. There was a positive, statistically significant relation between calprotectin and C-reactive protein values (*r* = 0. 292 *p* = 0. 001, respectively). There was no statistically significant difference was determined between calprotectin and white blood cell values (*r* = 0. 142 *p* = 0. 187, respectively). CRP and Cal values were significantly higher in patients with a complicated AA group than in those with uncomplicated AA (*p* = 0. 014, *p* = 0. 0001, respectively) whereas white blood cell counts did not differ significantly between two groups (*p* = 0. 164).

**Conclusion:**

Plasma calprotectin levels were increased in patients with acute appendicitis and should use in a distinction of uncomplicated from complicated acute appendicitis patients.

## Background

The lifetime occurrence of acute appendicitis (AA) is approximately 7–8 % and it is the most common disease, which find in emergency surgery [1, 2]. Although the most severe of all adverse events, mortality in developed health systems is low (between 0.09 % and 0.24 %) and does not have a sensitivity to detect differences in care processes that lead to variation in other outcomes [3, 4]. No inflammatory marker alone, such as white blood cell (WBC) count, C-reactive protein (CRP) or other novel tests, including procalcitonin, can identify AA with high specificity and sensitivity [5]. However, WBC count is obtained in virtually all patients who are assessed for AA, when available. A range of novel biomarkers has been suggested during the past decade, including bilirubin, but these do not have external validity and suffer repeatedly from low sensitivity, which means they are unlikely to come into clinical practice [6]. Initial reliance on ultrasound (USG) has become more guarded recently because of moderate sensitivity (86 %, CI 83–88) and specificity (81 %, CI 78–84) as shown through pooled diagnostic accuracy of 14 studies [7], limiting its diagnostic ability. In adolescent and adult patients, computed tomography (CT) has become the most widely accepted imaging strategy. It is used in 86 % of patients, with a sensitivity of 92 · 3 % [8]. The rate of negative appendectomy decreased significantly over time, from 12 · 7 % in 1995 to 2 · 8 % in 2006 [9–11].

Calprotectin (Cal) S-100 is a 36-kDa heterodimer that belongs to the family of calcium-binding proteins, which involve both lights (MRP8) and heavy (MRP14) chains and were identified as an antimicrobial protein in granules of neutrophils and to a lesser extent in monocytes and reactive macrophages [12]. Cal was counting 60 % of the total cytosolic protein in neutrophils [13], which provide to separate these from monocytes during cell death and cell rupture [12]. In the presence of an ongoing cycle of inflammation, Cal levels are increased in plasma, synovial fluid, urine and tool [14–16]. Thus, Cal levels in plasma and various body fluids have been proposed as a marker of inflammation [12].

In this prospective study, the authors have aimed to compare of inflammatory markers, which use in routine practice and plasma Cal values and to identify the role of plasma Cal values in the diagnosis of complicated and uncomplicated AA. Therefore, in this study, we measured plasma Cal and CRP levels in patients with AA to investigate the diagnostic accuracy of the combined use of these markers.

## Methods

The Strengthening the Reporting of Observational Studies in Epidemiology (STROBE) statement was used in the design and implementation of the study and to prepare the manuscript [17]. The study was approved by the Local Ethics Committee of our hospital (01-2013) and judged that informed consent from patients was not necessary because of the observational study design with no additional burden for the patient.

Eighty-nine patients with AA, with an ASA (American Society of Anesthesiologists) score of I–III, who underwent laparoscopic appendectomy in our general surgery clinic between January 2013 and May 2013 included the study. The control group consisted of 30 patients, which were selected from a group of 67 patients, who admitted to the emergency clinic with the complaint of abdominal pain in the right or right and left lower quadrants, after laboratory tests and abdominal imaging, necessary consultations were performed and follow-up for 48 h, during which time neither antibiotics nor non-steroidal anti-inflammatory drugs were administered, diagnostic laparoscopy was performed in these patients. In 18 of 67 patients were determined a pelvic inflammatory disease, 16 patients had hemorrhagic ovarian cyst or cyst rupture, and 3 patients had diverticulitis at laparoscopic exploration. Since Cal values of plasma have been increased with inflammatory process in these pathological cases and if patients in the control group have these pathologic findings, excluded the study. The control group was created from 30 patients without intra-abdominal pathology and thus diagnosed as non-specific abdominal pain.

A pilot study, which were conducted with 20 patients in each group. As a result of the study, the effect size for Cal values was determined to be 0.609. Three patients for each control group member were taken. With 0.05 Type I error rate, %80 power and allocation size of 3, the minimum needed numbers were determined as 29 and 87 for control group respectively.

At our hospital, the normal laboratory values for CRP and WBC are 0.01–0.5 mg/dL and 4–11 × 103/mm, respectively. The detection limit for Cal, according to Hycult Biotech is 46.8 ng/ml [18]. The Cal values of the AA group were compared with the control group, which were in normal range. Blood samples were centrifuged for 15 min at 2000 × g. Aliquots of plasma were stored at–80 °C until used in the assays. Serum Cal was determined using a human Cal ELISA kit (East Biopharm, China) according to the manufacturer’s instructions. Cal level was expressed ng/ml. The limit of detection of the ELISA is 20 ng/mL. The intra-assay and inter-assay coefficients of variation were < 8.1 % and <7.6 %, respectively.

Cal was measured in blood samples obtained from both the AA and control groups. To patients, who presented with abdominal pain, a physical examination, laboratory tests and abdominal USG and CT were performed. In the AA group, blood samples for Cal, WBC and CRP measurements were obtained after diagnosis and before medical and surgical treatment. The abdominal cavities of these patients were explored during the laparoscopic appendectomy. Only those without any additional intra-abdominal inflammatory pathologies were included the study. Complicated AA was defined as AA in which perforation, gangrenous or an intra-abdominal abscess [19].

### Statistical analysis

Statistical analysis was performed using the Statistical Software Package Program (Utah, USA), NCSS (Number Cruncher Statistical System) 2007. When evaluating data, besides routine statistical analysis, such as mean, standard deviation, median, frequency and rate, in the intergroup comparison of variables with normal distribution, Independent Sample test and in the intergroup comparison of variables in the normal distribution Mann-Whitney *U* test were used. Yates’ continuity correction test (Yates corrected chi-square) was used in the comparison of qualitative data. Cut-off values for Call were assessed using ROC curves. The results were assessed within a confidence interval of 95 % and significance was assessed at *p* < 0.05 level.

## Results

The male-to-female ratio was 54/35 in the study of patients and 20/10 in the control patients. The mean age was 28 (range 19–45) and 31 (range 21–56), respectively. AA was histopathologically confirmed in 89 patients and determination of acute mucosal inflammation was in 22 patients (24.7 %), AA with phlegmon formation in 48 patients (53.9 %), and AA with gangrene in 19 patients (21.3 %). A ruptured appendix was detected in 18 of 89 cases with AA. Findings of peritonitis and evaluation of appendix microscope with histopathological results have shown, 23 (26 %) of patients had complicated and 66 (74 %) of patients had uncomplicated AA.

WBC levels were significantly higher in the AA group than in the control group (*p* = 0. 0001), as high as in CRP (*p* = 0. 0001) and Cal (*p* = 0. 0001) levels. The relationship between Cal and CRP values was statistically significant (*p* = 0.001, *r* = 0.292), whereas this was not the case for Cal and WBC values (*p* = 0.187, *r* = 0.141) (Table 1). Average plasma Cal values were 59 ng/ml (range 46–107) in the group of patients with acute mucosal inflammation, 68 ng/ml (45–109) in those with phlegmonous AA, and 185 ng/ml (range 85–538) in those with gangrenous or ruptured AA. CRP and Cal values were significantly higher in patients with a complicated AA group than in those with uncomplicated AA (*p* = 0. 014 and *p* = 0. 0001, respectively) whereas WBC counts did not differ significantly between two groups (*p* = 0. 164) (Table 2). However, WBC, CRP and Cal values were significantly higher in patients with positive USG findings [USG (+)], than in those AA patients with negative USG findings [USG (−)] with AA (*p* = 0. 0001) (Table 3).

**Table 1:** WBC, CRP and Cal values in the AA and control group

		AA	Control	*p*
WBC	Mid ± SS	14,11 ± 5,62	7,21 ± 3,79	0,0001
	Median (IQR)	13,9 (10,9–17,3)	6,75 (5,9–7,9)	
CRP	Mid ± SS	5,92 ± 5,48	0,50 ± 0,42	0,0001
	Median (IQR)	4,2 (2,14–7,7)	0,40 (0,26–0,66)	
Cal	Mid ± SS	91,56 ± 77,40	47,18 ± 32,55	0,0001
	Median (IQR)	67,12 (53,87–67,12)	39,50 (34,81–42,71)	
		Cal		
CRP	r	0,292		
	p	0,001		
WBC	r	0,141		
	p	0,187

**Table 2:** WBC, CRP and Cal values in the uncomplicated and complicated AA groups

		Uncomplicated AA	Complicated AA	*p*
WBC	Mid ± SS	5,78 ± 5,6	6,43 ± 5,15	0,164
	Median (IQR)	3,78 (1,75–8,28)	6,43 ± 5,15	
CRP	Mid ± SS	13,54 ± 4,74	16,21 ± 3,52	0,014
	Median (IQR)	13,35 (10,4–16,93)	16,21 ± 3,52	
Cal	Mid ± SS	65,44 ± 17,12	187,78 ± 125,45	0,0001
	Median (IQR)	59,58 (52,29–72,75)	187,78 ± 125,45

**Table 3:** WBC, CRP and Cal values in the USG (+/−)

		USG (−)	USG (+)	*p*
WBC	Mid ± SS	13,85 ± 4,54	14,31 ± 4,71	0,0001
	Median (IQR)	14,75 (10,75–17,3)	13,60 (10,8–18,2)	
CRP	Mid ± SS	7,38 ± 6,31	4,83 ± 4,54	0,0001
	Median (IQR)	5,5 (2,7–9,77)	3,75 (1,76–5,93)	
Cal	Mid ± SS	90,46 ± 87,9	92,37 ± 69,45	0,0001
	Median (IQR)	63,62 (52,3–98,42)	68,5 (55,57–97,1)	

**Fig. 1:**
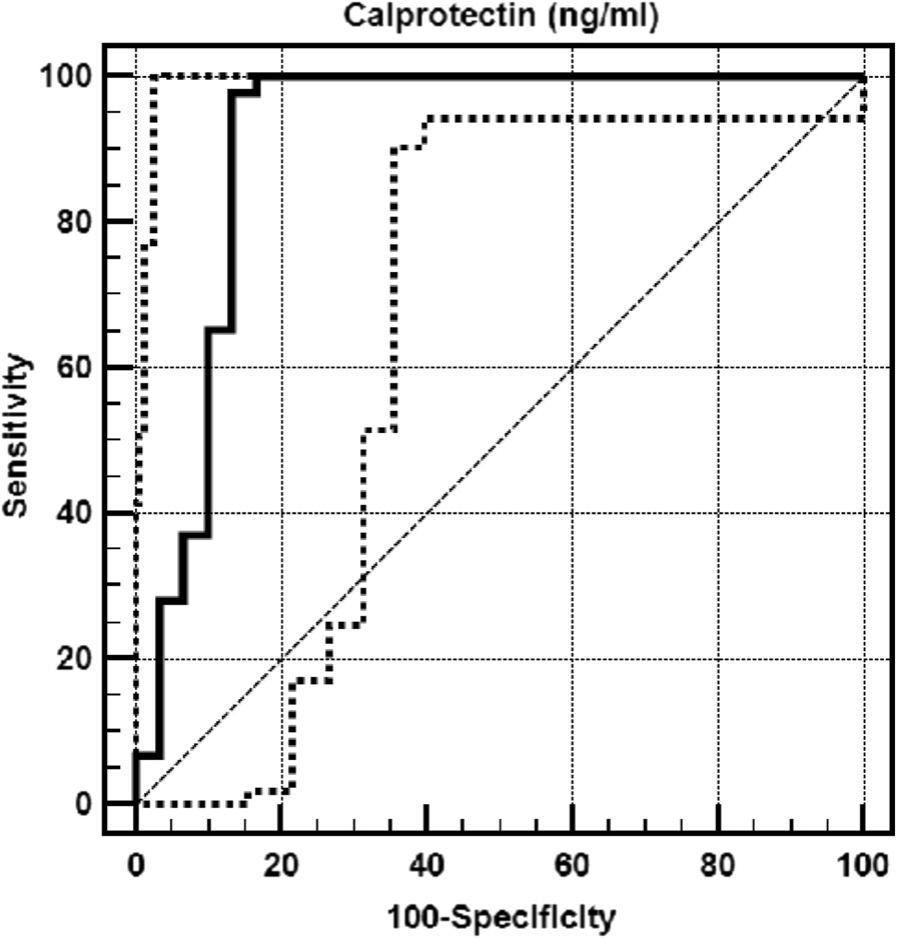
ROC curve, specificity and sensitivity for Cal

In terms of the presence of AA, statistically significant differences (*p* = 0. 001; *p* < 0.01) was observed between the values of Cal, and the results clearly had showed that Cal level is high if AA is the presence. Following these results, it has been decided to study the cut-off point for Cal. In terms of the presence of AA, ROC analyzes and the full-screen test were used to determine cut-off point. In terms of the presence of AA, it has been observed that the cut-off point for Cal is 46 and higher. For the 46 ng/mL cutoff value of Cal; sensitivity is %98. 88, specificity is %83. 33, positive cutoff value 94.62 and negative cutoff value is 96.15. On the ROC curve, the standard error for the below part %91. 2 is %4. 3 (Fig. 1).

**Fig. 2:**
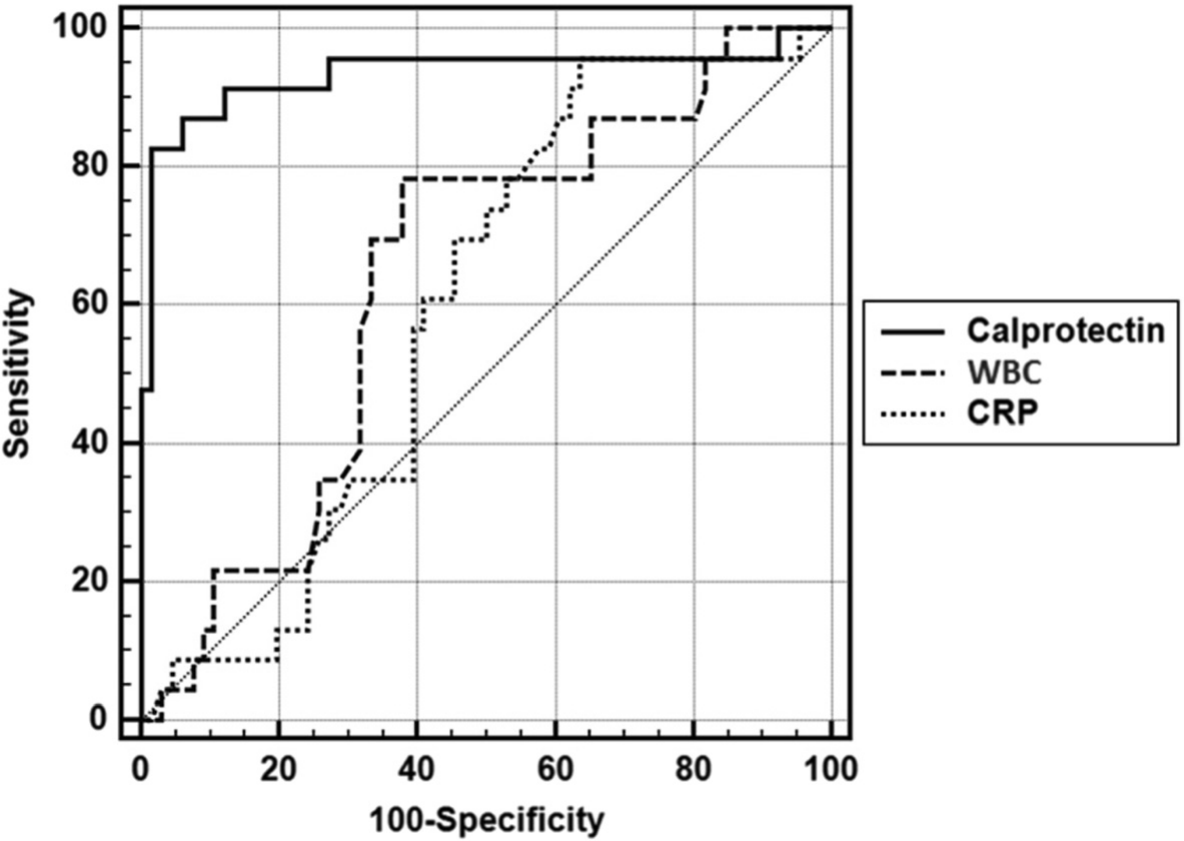
ROC curve for Cal, WBC and CRP

Statistically significant relation between the presence of AA and 46 cut-off value of Cal was observed (*p* = 0.001; *p* < 0.01). When Cal level is 46 and higher, the risk to see AA is 7.33 times more. While the below part of the ROC curve is 0.912 for Cal, it is 0.952 for WBC and 0.824 for CRP. Significant differences in the below ROC curve was observed (*p* = 0. 942; *p* = 0. 952; *p* = 0. 824; p > 0.05, respectively). (Table 4) If leukocyte, CRP and Cal values below the ROC curve were evaluated, value for Cal was 0.935 below ROC curve, 0.640 for WBC and 0.597 for CRP and values below the ROC curve was statistically significant (*p* < 0.01) (Fig. 2). These findings helped us to find the value of Cal in complicated AA was more effective than WBC and CRP values (*p* = 0.001; *p* = 0.001; *p* < 0.01, respectively). Any significant difference wasn’t determined between WBC and CRP in pre-diagnosis of complicated AA (*p* = 0. 603; p > 0.05) (Table 5).

**Table 4:** The area under the ROC curve (AUC) for Cal, WBC and CRP in the AA

	AUC	SE	%95 Cl
Cal	0,912	0,044	0,846–0,956
WBC	0,909	0,027	0,842–0,954
CRP	0,914	0,026	0,849–0,958
Cal- WBC	p= 0,942		
Cal-WBC	p = 0,952		
WBC -CRP	p = 0,824

**Table 5:** The area under the ROC curve (AUC) for Cal, WBC and CRP in the uncomplicated and complicated AA groups

	AUC	SE	%95 Cl
Calprotectin	0,935	0,042	0,862–0,976
WBC	0,640	0,064	0,532–0,739
CRP	0,597	0,062	0,488–0,700
Calprotectin- WBC	p = 0,001		
Calprotectin-CRP	p = 0,001		
WBC -CRP	p = 0,603		

## Discussion

There is currently no evidence for suggesting, that serum Cal is superior to standard inflammatory markers for the exclusion or confirmation of suspected AA [20]. However, uncomplicated and complicated AA is considered to be two entities. The curability of uncomplicated disease without surgery is proven in studies comparing to antibiotic therapy, whereas complicated AA with necrosis or perforation of the appendix cannot be treated successfully without invasive modality. The preferred surgical approach in complicated AA is more unclear, therefore of lack of evidence in this group [21]. In this study has supported, that plasma CRP level increase was nonspecific, as in literature. Another fact that, we wanted to emphasize the identification of Cal was a useful inflammatory parameter. In distinction of complicated and uncomplicated AA, Cal was statistically more effective than the other inflammatory parameters according to imaging and laboratory methods [21]. When inflammatory process progresses and peritonitis findings have occurred, Cal level has also increased. Due to thickening of appendix wall, inflammation around fat plans or the liquid amount around the appendix, plasma Cal level has increased, and, therefore, Cal levels were determined USG (+) patients higher than USG (−) patients [21].

In this study, the average plasma Cal value of the control group was 47.18 ng/ml, which was close to the value, which was reported by Hycult Biotechnology (46.8 ng/ml). Cal levels in AA group were significantly higher than the control group. Although this increase was non-specific for AA, it was specifically for the acute inflammatory process, as well as CRP value, which has a plasma half-life of 19 h and is produced by the liver [22–25]. The relationship between plasma Cal and CRP levels was statistically meaningful.

Cal was an acute-phase reactant, which increases in local and systemic inflammatory diseases [23]. According to Yui et al. This increase was caused by the migration of leukocytes in the region of inflammation and tissue disruption [26, 27]. Thus, in colorectal cancer, inflammatory bowel disease, necrotizing enterocolitis and celiac diseases fecal Cal levels have increased. According to this, fecal Cal has been proposed as a non-invasive, non-specific marker for the diagnosis of these diseases and for detection of disease activation and remission in patients with ulcerative colitis and Crohn’s Disease [27–32]. In AA, increased plasma Cal levels, when were used together with WBC and CRP levels might allow a more accurate diagnosis of AA [33, 34]. While plasma Cal levels also increase in acute pancreatitis, whether similar increases could be measured in the pancreatic inflammation included further study [35].

Despite the use of laboratory tests, imaging techniques and clinical examinations in patients with suspected AA, both the number of negative laparotomy/laparoscopy and surgery-related morbidity and mortality were remaining high [36, 37]. On the other hand, routine diagnostic laparoscopy has been proposed as an alternative to in-hospital observation in patients with suspicion of AA [10]. Two recent reviews of randomized trials, comparing early laparoscopy to observation did not perform, however, determine not any clear advantage [38, 39]. Investigations to help distinguish those patients in the mid-Alvarado Score group includes CRP, USG and CT. CRP as a blood test for AA has a relatively high specificity: according to this, a patient with normal CRP level is unlikely to have AA [5]. Compared to WBC and procalcitonin, a meta-analysis determined, CRP was more accurate [5]. One study recommends that Cal, CRP or WBC count did not have high sensitivity and specificity to be clinically useful in the evaluation of subjects with suspected AA. Likewise, procalcitonin had little effect in diagnosing AA, with lower diagnostic accuracy than CRP and WBC [40].

The diagnosis of AA depends on the patient’s history, a detailed physical examination, completed with laboratory tests and imaging methods. In patients with suspected AA, together elevated CRP levels and increased plasma Cal levels could provide further support for the diagnosis. Whether the use of CRP and Cal levels in the diagnosis of AA, decreased the rate of unnecessary and delayed laparotomies/laparoscopies remains to be determined in larger groups of patients.

Our study has several limitations, such as a low number of patients and was a single-center design. This study was prospective, but the study design and data analysis were performed retrospectively. Once the diagnosis was confirmed and the study group was selected retrospectively. There is a lack of validation cohort in this study.

## Conclusion

Plasma Cal levels could be used indistinctness of uncomplicated from complicated acute appendicitis as a diagnostic marker of acute appendicitis.

## References

[1] Jacobs JE (2006). CT and sonography for suspected acute appendicitis: a commentary. AJR Am J Roentgenol.

[2] Bhangu A, Søreide K, Di Saverio S, Assarsson JH, Drake FT (2015). Acute appendicitis:modern understanding of pathogenesis, diagnosis, and management. Lancet.

[3] Bliss LA, Yang CJ, Kent TS, Ng SC, Critchlow JF, Tseng JF (2014). Appendicitis in the modern era: universal problem and variable treatment. Surg Endosc.

[4] Faiz O, Clark J, Brown T (2008). Traditional and laparoscopic appendectomy in adults: outcomes in English NHS hospitals between 1996 and 2006. Ann Surg.

[5] Yu CW, Juan LI, Wu MH, Shen CJ, Wu JY, Lee CC (2013). Systematic review and meta-analysis of the diagnostic accuracy of procalcitonin, C-reactive protein and White blood cell count for suspected acute appendicitis. Br J Surg.

[6] Andersson M, Ruber M, Ekerfelt C, Hallgren HB, Olaison G, Andersson RE (2014). Can new inflammatory markers improve the diagnosis of acute appendicitis?. World J Surg.

[7] Terasawa T, Blackmore CC, Bent S, Kohlwes RJ (2004). Systematic review: computed tomography and ultrasonography to detect acute appendicitis in adults and adolescents. Ann Intern Med.

[8] Cuschieri J, Florence M, Flum DR (2008). Negative appendectomy and imaging accuracy in the Washington State Surgical Care and Outcomes Assessment Program. Ann Surg.

[9] Van Rossem CC, Bolmers MD, Schreinemacher MH, van Geloven AA, Bemelman WA, Snapshot Appendicitis Collaborative Study Group (2015). Prospective nationwide outcome audit of surgery for suspected acute appendicitis. Br J Surg.

[10] Andersson RE (2014). Short-term complications and long-term morbidity of laparoscopicand open appendectomy in a national cohort. Br J Surg.

[11] Güller U, Rosella L, McCall J, Brügger LE, Candinas D (2011). Negative appendectomyand perforation rates in patients undergoing laparoscopic surgery for suspected appendicitis. Br J Surg.

[12] Dale I, Fagerhol MK, Naesgaard I (1983). Purification and partial characterization of highly immunogenetic human leukocyte protein, the L1 antigen. Eur J Biochem.

[13] Stockley RA, Dale I, Hill SI, Fagerhol MK (1984). Relationship of neutrophil cytoplasmic protein (L1) to acute and chronic lung disease. Scand J Clin Lab Invest.

[14] Ton H, Brandsnes, Dale S, Holtlund J, Skuibina E, Schjønsby H (2000). Improved assay for fecal Cal. Clin Chim Acta.

[15] Konikoff MR, Denson LA (2006). Role of fecal Call as a biomarker of intestinal inflammation in inflammatory bowel disease. Inflamm Bowel Dis.

[16] Roseth AG, Aadland E, Jhansen J, Raknerud N (1997). Assessment of disease activity in ulcerative colitis by fecal Cal, a novel granulocyte marker protein. Digestion.

[17] von Elm E, Altman DG, Egger M, Pocock SJ, Gotzsche PC, Vandenbroucke JP (2007). The Strengthening the Reporting of Observational Studies in Epidemiology (STROBE) statement: guidelines for reporting observational studies. Lancet.

[18] Hanssen SJ, Derikx JP, VermeulenWindsant IC, Heijmans JH, Koeppel TA, Schurink GW (2008). Visceral injury and systemic inflammation in patients undergoing extracorporeal circulation during aortic surgery. Ann Surg.

[19] Al-Omran M, Mamdani MM, McLeod RS (2003). Epidemiologic features of acute appendicitis in Ontario, Canada. Can J Surg.

[20] Horner D, Long AM (2013). Towards evidence-based emergency medicine: best BETs fromthe Manchester Royal Infirmary. BET 3: Super calprotectin will not expedite yourdischarge. Emerg Med J.

[21] Atema JJ, van Rossem CC, Leeuwenburgh MM, Stoker J, Boermeester MA (2015). Scoring system to distinguish uncomplicated from complicated acute appendicitis. Br J Surg.

[22] Matthiessen P, Henriksson M, Hallböök O, Grunditz E, Norén B, Arbman G (2008). Increase of serum C-reactive protein is an early indicator of subsequent symptomatic anastomotic leakage after anterior resection. Colorectal Dis.

[23] Mustard RA, Bohnen JM, Haseeb S, Kasina R (1987). C-reactive protein levels predict postoperative septic complications. Arch Surg.

[24] Welsch T, Müller SA, Ulrich A, Kischlat A, Hinz U, Kienle P (2007). C-Reactive protein as early predictor for infectious postoperative complications in rectal surgery. Int J Colorectal Dis.

[25] Golden BE, Clohessy PA, Russell G, Fagerhol MK (1996). Calprotectin as a marker of inflammation in cystic fibrosis. Arch Dis Child.

[26] Yui S, Mikami M, Yamazaki M (1995). Induction of apoptotic cell death in Mouse lymphoma and human leukemia cell lines by a calcium-binding protein complicated, calprotectin, derived from inflammatory peritoneal exudate cells. J Leuk Biol.

[27] Stritz I, Trebichavsky I (2004). Calprotectin - a pleiotropic molecule in acute and chronic inflammation. Physiol Res.

[28] Zhao L, Wang H, Sun X, Ding Y (2010). Comparative proteomic analysis identifies proteins associated with the development and progression of colorectal carcinoma. FEBS J.

[29] Burri E, Beglinger C (2012). Faecal calprotectin - a useful tool in the management of in flammatory bowel disease. Swiss Med Wkly.

[30] Aomatsu T, Yoden A, Matsumoto K, Kimura E, Inoue K, Andoh A (2011). Fecalcalprotectin is a useful marker for disease activity in pediatric patients with in flammatory bowel disease. Dig Dis Sci.

[31] Ho GT, Lee HM, Brydon G, Ting T, Hare N, Drummond H (2009). Fecal calprotectin predicts the clinical course of acute severe ulcerative colitis. Am J Gastroenterol.

[32] Jensen MD, Kjeldsen J, Nathan T (2011). Fecal calprotectin is equally sensitive in Crohn’s disease affecting the small bowel and colon. Scand J Gastroenterol.

[33] Thuijls G, Derikx JP, Prakken FJ, Huisman B, van BijnenIng AA, van HeurnEL BWA (2011). A pilot study on potential new plasma markers for diagnosis of acute appendicitis. Am J Emerg Med.

[34] Bealer JF, Colgin M (2010). S100A8/A9: a potential new diagnostic aid for acute appendicitis. Acad Emerg Med.

[35] Carroccio A, Rocco P, Rabitti PG, Di Prima L, Forte GB, Cefalù AB (2006). Plasma calprotectin levels in patients suffering from acute pancreatitis. Dig Dis Sci.

[36] Sugi K, Saitoh O, Hirata I, Katsu K (1996). Fecal lactoferrin as a marker for disease activity in inflammatory bowel disease: comparison with other neutrophil-derived proteins. Am J Gastroenterol.

[37] Lee SL, Walsh AJ, Ho HS (2001). Computed tomography and ultrasonography do not improve and may delay the diagnosis and treatment of acute appendicitis. Arch Surg.

[38] Domínguez LC, Sanabria A, Vega V, Osorio C (2011). Early laparoscopy for the evaluation of nonspecific abdominal pain: a critical appraisal of the evidence. Surg Endosc.

[39] Maggio AQ, Reece-Smith AM, Tang TY, Sadat U, Walsh SR (2008). Early laparoscopy versus active observation in acute abdominal pain: systematic review and meta-analysis. Int J Surg.

[40] Schellekens DH, Hulsewé KW, van Acker BA, van Bijnen AA, de Jaegere TM, Sastrowijoto SH (2013). Evaluation of the diagnostic accuracy of plasma markers for early diagnosis in patients suspected for acute appendicitis. Acad Emerg Med.

